# Identification of two contiguous minimally deleted regions on chromosome 1p36.31–p36.32 in oligodendroglial tumours

**DOI:** 10.1038/sj.bjc.6602093

**Published:** 2004-09-10

**Authors:** Z Dong, JC-S Pang, M H Ng, W S Poon, L Zhou, H-K Ng

**Affiliations:** 1 Department of Anatomical and Cellular Pathology, Prince of Wales Hospital, The Chinese University of Hong Kong, Hong Kong, China; 2 Neurosurgical Unit, Department of Surgery, Prince of Wales Hospital, The Chinese University of Hong Kong, Hong Kong, China; 3 Department of Neurosurgery, Hua Shan Hospital, Fudan University, Shanghai, China

**Keywords:** oligodendroglioma, oligoastrocytoma, tumour suppressor gene, loss of heterozygosity, chromosome 1p

## Abstract

Loss of the short arm of chromosome 1 is a hallmark of oligodendroglial tumours (OTs). Deletion mapping studies in OTs have revealed multiple commonly deleted regions on chromosome 1p, suggesting that there are more than one tumour suppressor gene. To map critical deletion regions on 1p, a series of 25 OTs were examined for loss of heterozygosity (LOH) on 19 polymorphic markers across the 1p arm using microsatellite analysis. Our study revealed that 60% of tumours had LOH of all informative markers on 1p and identified one tumour showing LOH at telomeric markers only. Since this deletion region lies in one of the critical deletion intervals defined previously, we then screened another series of 27 OTs specifically at 1p36.3 for LOH using nine polymorphic markers. A total of 12% (six out of 52) of tumours were found to carry interstitial deletions. The allelic status and the deletion breakpoints in these tumours with interstitial deletion were further verified by fluorescent *in situ* hybridisation. The small overlapping intervals facilitated the delineation of two contiguous minimally deleted regions of 0.76 Mb, defined by D1S468 and D1S2845, and of 0.41 Mb, bound by D1S2893 and D1S1608, on 1p36.31–36.32. Based on current reference human genome sequence these deletion regions have been sequenced almost to entirety and contain eight annotated genes. TP73, DFFB and SHREW1 are the only known genes located in these deletion regions, while the others are uncharacterised novel genes. In conclusion, our study has narrowed down the critical tumour suppressor loci on 1p36.3, in which two minimally deleted regions are mapped, and markedly reduced the number of candidate genes to be screened for their involvement in OT development.

Oligodendroglial tumours (OTs) are primary brain neoplasms that constitute about 5–18% of all gliomas. These tumours comprise the classic oligodendrogliomas and mixed oligoastrocytomas. Two malignancy grades are recognised by the World Health Organization (WHO): grade II for the well-differentiated tumours and grade III for the anaplastic variants (Reifenberger *et al*, 2000). Unlike other glioma types, OTs have a unique clinical feature of remarkable responsiveness to chemotherapy (Macdonald *et al*, 1990; Perry *et al*, 1999).

The pathogenesis of OTs is poorly understood. Molecular studies have shown that allelic deletions of chromosome arms 1p and 19q are the most frequent genetic alterations in OTs, suggesting that these chromosomes carry critical tumour suppressor genes and their inactivation is involved in the early stage of OT development (Bello *et al*, 1995). The frequencies of allelic loss of 1p and 19q in OTs are 67–94 and 70–76%, respectively, and of combined losses is 64–72% (Bello *et al*, 1995; Bigner *et al*, 1999; Husemann *et al*, 1999; Jeuken *et al*, 1999; Smith *et al*, 1999; Iuchi *et al*, 2002). Other genetic alterations occurring at lower frequencies include losses of chromosomes 4, 9, 10, 14, 15 and 18 (Bigner *et al*, 1999; Jeuken *et al*, 1999). With regard to target genes, the CDKN2A tumour suppressor gene located on 9p21 is found to be homozygously deleted in about 25% of anaplastic oligodendrogliomas (Cairncross *et al*, 1998; Bigner *et al*, 1999; Watanabe *et al*, 2001a) and the PTEN gene maps to 10q23 is mutated in 8% of anaplastic oligodendrogliomas (Sasaki *et al*, 2001). In addition, recent studies have shown that epigenetic change also plays an important role in OT formation. Promotor hypermethylation of RB1 and p14^ARF^ is detectable, respectively, in 34% (Dong *et al*, 2001; Watanabe *et al*, 2001b) and 18–41% (Watanabe *et al*, 2001a, Wolter *et al*, 2001; Alonso *et al*, 2003) of OTs. Taken together, these results suggest that at least three genetic pathways involving CDKN2A/RB1, p14^ARF^/p53 and PTEN/PI3K-AKT are dysregulated in OT development. On contrary, the target genes from the frequently deleted chromosome arms 1p and 19q are not yet identified.

The majority of OTs harbouring 1p deletion show allelic loss in all informative markers, indicating that entire or most of short arm has been deleted. Hence, the deletion breakpoints on 1p have been derived from a limited number of tumours carrying small deletions (Reifenberger *et al*, 1994; Bello *et al*, 1995; Zhu *et al*, 1998b; Husemann *et al*, 1999; Smith *et al*, 1999; Iuchi *et al*, 2002). Husemann *et al* (1999) identified two distinct regions of loss from three OTs carrying interstitial deletions: a distal region between D1S76 and D1S253 at 1p36.3 (∼5 Mb) and a proximal region between D1S482 and D1S2743 (17 Mb) at 1p34.2–p36.12. Smith *et al* (1999) mapped a commonly deleted region to 1p36.31–p36.32 (3.7 Mb) between D1S468 and D1S1612, whereas Iuchi *et al* (2002) delineated two regions of loss to 1p34–p35 (∼5.7 Mb) and 1p36.1–p36.2 (∼12 Mb). These deletion regions do overlap and may be refined to three loci: 1p36.3 (between D1S468 and D1S253 of ∼2.9 Mb), 1p36.1 (between D1S482 and D1S1676 of 1.4 Mb) and 1p34.3–p35 (between D1S247 and D1S496 of 4.4 Mb) (Figure 1

**Figure 1:**
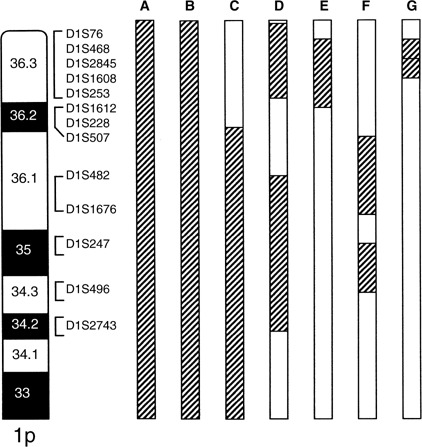
Deletion mapping studies of chromosomal arm 1p in OTs. (**A**) Reifenberger *et al* (1994); (**B**) Bello *et al* (1995); (**C**) Zhu *et al* (1998b); (**D**) Husemann *et al* (1999); (**E**) Smith *et al* (1999); (**F**) Iuchi *et al* (2002); (**G**) current study. Striped bars represent minimally deleted regions identified in each study.

). The identification of multiple deletion regions suggests that there is more than one tumour suppressor gene on the 1p arm. The possibility of multiple tumour suppressor loci on 1p has also been suggested from studies of other tumour types such as meningiomas (Bello *et al*, 2000b). Two candidate genes, CDKN2C (1p32.3) and hRAD54 (1p34.1–p33) were evaluated for their involvement in OT. Somatic mutation and homozygous deletion of CDKN2C was detected in 2–5% anaplastic oligodendrogliomas, suggesting alteration of this gene is involved in progression of a subset of tumours (Husemann *et al*, 1999; Pohl *et al*, 1999; He *et al*, 2000). No base alterations were, however, found in hRAD54 (Bello *et al*, 2000a).

Recent studies have also demonstrated that allelic loss of 1p is closely associated with chemosensitivity and better prognosis in patients with anaplastic oligodendrogliomas (Cairncross *et al*, 1998; Ino *et al*, 2001). Thus, investigation into the basis of 1p loss would unveil not only the pathogenesis of OT, but might also provide insights into the genetic origin of these clinical responses. In this study, we reported the refinement of one of the critical deletion regions in OT to 1p36.31–36.32 and the mapping of two contiguous minimally deleted regions, in which there are only eight genes annotated.

## MATERIALS AND METHODS

### Tumour specimens and DNA extraction

A cohort of 52 OTs, including 22 oligodendrogliomas (WHO grade II), 14 anaplastic oligodendrogliomas (WHO grade III), 13 oligoastrocytomas (WHO grade II) and three anaplastic oligoastrocytomas (WHO grade III), and their corresponding blood samples were obtained from Prince of Wales Hospital, Hong Kong and Hua Shan Hospital, Shanghai, China. The OTs were classified according to the current WHO Classification of Tumours of Nervous System (Reifenberger *et al*, 2000). Histological assessment of tissue sections confirmed a tumour cell content of at least 80% in all samples. The average age of patients was 40.8±15.3 years (range, 8–74) and the male/female ratio was 1.4 : 1.

DNA was prepared from tissue and blood specimens using the conventional proteinase K digestion and phenol/chloroform extraction method. DNA extracted from blood leukocytes was used as constitutional control.

### Microsatellite analysis

A total of 30 microsatellite loci, located on chromosome arms 1p, 1q and 19q, were investigated for loss of heterozygosity (LOH) according to reported protocol (Tong *et al*, 2001). In all, 26 polymorphic markers map to 1p arm (D1S171, D1S468, D1S2845, D1S2893, D1S2660, D1S1608, D1S2132, D1S2795, D1S2870, D1S1646, D1S1612, D1S2667, D1S2647, D1S482, D1S470, D1S496, D1S2743, D1S2724, D1S2752, D1S473, D1S1665, D1S2807, D1S188, D1S2808, D1S2695 and D1S514), two markers map to 1q arm (D1S215 and D1S2625) and two markers locate to chromosome 19q (D19S219 and D19S412). These markers were labelled either with fluorochrome FAM or HEX (Proligo, Singapore). Briefly, polymerase chain reaction (PCR) was performed in a final volume of 5 *μ*l containing 50–100 ng of DNA, 10 mM Tris-HCl (pH 8.3), 50 mM KCl, 2.5 mM MgCl_2_, 0.2 mM deoxyribonucleoside triphosphates, 0.5 U of AmpliTaq Gold DNA polymerase (Applied Biosystems, Foster City, USA), and 0.4 *μ*M of each primer. The PCR commenced with an enzyme activation step at 95°C for 10 min, 40 cycles of 94°C for 1 min, 55–60°C for 1 min and 72°C for 1 min, and finalised with an elongation step at 72°C for 10 min. PCR products of multiple markers were pooled and electrophoresed in denaturing 4% polyacrylamide gels using an ABI Prism 377 automated DNA sequencer (Applied Biosystems). The data collected were analysed using GeneScan Analysis software version 3.1 (Applied Biosystems). Allelic imbalance was defined by calculating the allelic ratio of both normal (N) and tumour (T) DNA, based on the formula of (N2/N1)/(T2/T1), where N2 or T2 represents the peak height of the longer allele and N1 or T1 represents the peak height of the shorter allele. LOH was inferred when the allelic ratio was greater than 1.5 or smaller than 0.5, representing loss of the shorter or longer allele, respectively.

### FISH

Dual-colour FISH analysis was performed on formalin-fixed paraffin-embedded tissue sections as reported with modification (Jenkins *et al*, 1997). Section of 5-*μ*m thickness were deparaffinised, dehydrated, treated with sodium thiocyanate at 80°C for 10 min, digested in pepsin solution (5 mg ml^−1^ in 0.2 N HCl) for 20–30 min, rinsed in phosphate-buffered saline and dehydrated. The sections were then treated with microwave (600 W) in citrate buffer (pH 6.0) for 10 min, baked at 80°C for 30 min and subjected to hybridisation.

Seven bacterial artificial chromosomes (BACs) or bacteriophage P1-derived artificial chromosomes (PACs) were labelled to generate locus-specific FISH probes. These included six target probes for 1p arm and one reference probe for 1q32 (BAC clone RP11-79I9 encompassing the marker STSG28964). The genomic clones were purchased from Invitrogen Corporation (Carlsbad, USA). DNA was labelled by nick-translation in a reaction containing 100 mM Tris-HCl (pH 7.5), 10 mM MgCl_2_, 0.01% bovine serum albumin, 0.1 mM dATP, 0.1 mM dCTP, 0.1 mM dGTP, 0.1 nM dTTP, 0.1 nM *β*-mercaptoethanol, 0.03 U DNase I, 20 U of DNA polymerase I, and 0.85 nmol of digoxigenin-1-dUTP (for target probe) or 1.9 nmol of biotin-16-dUTP (for reference probe) (Roche Diagnostics Ltd., Hong Kong) at 15°C for 25–45 min. The labelled probes were mixed with fish sperm and Cot-1 DNA in Hybrisol VI solution (Appligene Oncor, Illkirch Graffenstaden, France), denatured and applied onto sections. Hybridisation was carried out at 37°C overnight. Sections were washed with 1.5 M urea/1 × saline sodium citrate (SSC) at 45°C for 30 min, 2 × SSC at 45°C for 15 min, treated with either antidigoxigenin-rhodamine antibody (Sigma) or avidin conjugated-fluorescein isothiocyanate (Vector Laboratories, Burlingame, USA) at 37°C for 1.5 h, washed in phosphate-buffered saline detergent (Appligene Oncor), and counterstained with Vectashield mounting medium with anti-fade solution of 4,6-diamidino-2phenylindole (DAPI; Vector Laboratories).

Sections were viewed under a Zeiss Axioplan microscope and images of rhodamine, FITC and DAPI were captured through a triple-pass filter by a cooled charge-coupled device. A total of 200 nonoverlapping tumour nuclei were counted on each section. A sample was considered having deletion of 1p locus when more than 70% of counted nuclei exhibited one target signal (red) and two reference signals (green).

### Statistical analysis

Statistical analysis was performed using the software SPSS 10.0. The correlation between two parameters was evaluated by *χ*^2^-test or Fisher's exact test, whichever was appropriate. An obtained *P*-value less than 0.05 (two-sided) was considered statistical significant.

## RESULTS

### Deletion mapping on chromosome 1p

**Figure 2:**
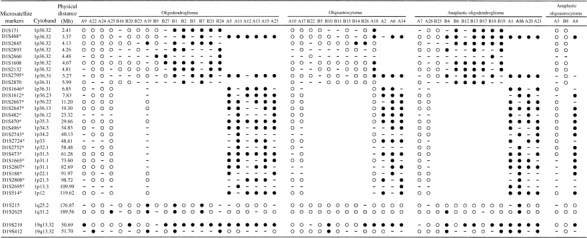
Summarised results of microsatellite analysis of 52 OTs examined. Cytoband and physical distance of microsatellite markers were derived from current reference human genome sequence (Build 34). The asterisks represent markers examined in the first round of microsatellite analysis. Filled circle=LOH; open circle=retention of heterozygosity; −=noninformative.

To localise tumour suppressor loci on the short arm of chromosome 1 in OTs, we carried out a deletion mapping study across the entire 1p arm using microsatellite analysis. A series of 25 OTs were investigated for LOH at 19 microsatellite loci, spacing at an average interval of 7 Mb on the 1p arm. Each tumour showed informativeness in at least seven markers. Overall, 15 (60%) tumours demonstrated LOH in at least one marker, with 14 of them showing LOH at all informative markers tested suggesting that these tumours had lost a copy of the entire or most of 1p arm. One tumour (A18) showed LOH at D1S468 and its juxtaposed marker D1S2795, both of which map to the telomeric end of 1p36.3, but retained heterozygosity at all informative markers centromeric to D1S2795 (Figure 2

). This deletion segment lies in the overlapped region of loss defined by Smith *et al* (1999) and Husemann *et al* (1999), indicating that 1p36.3 is a critical region involved in OT development. Since tumours with small chromosomal deletion are useful in delineation of tumour suppressor loci, we subsequently characterised another series of OTs focusing specifically at the 1p36.3 region with an aim to obtain additional cases harbouring interstitial deletion. We evaluated the allelic status of a total of nine (with seven additional new markers) polymorphic markers on 1p36.3 in a second series of 27 OTs and also in those tumours of the first series that showed small terminal deletion (i.e. case A18) or no LOH. The average interval between these nine markers is 0.46 Mb. In the second series, each tumour showed informativeness in at least three markers. Totally, 17 (63%) OTs demonstrated LOH in at least one marker, with 13 of them showing LOH at all informative markers. Four tumours (B9, B14, B26 and B27) exhibited LOH patterns suggestive of interstitial deletion. Cases B9, B14 and B26 lost heterozygosity at single marker, D1S2660 or D1S2845, while retaining balanced alleles at other informative loci. B27 showed LOH at two contiguous markers, D1S2660 and D1S1608, and maintained allelic balance at D1S2795, which is 0.69 Mb proximal to D1S1608. Other markers were noninformative in this tumour. In addition, our finer mapping has identified one more tumour (A19) with interstitial deletion from the initial set of tumours that showed no LOH. A19 had lost heterozygosity at D1S2845 on 1p. Surprisingly, A18 which initially displayed a telomeric deletion at 1p showed a zebra LOH pattern upon finer mapping. LOH was demonstrated at distal markers D1S171 and D1S468 and at proximal markers from D1S1608 to D1S2795, while allelic balanced markers were detected between these two regions and centromeric to D1S2795. This result suggests that A18 harbours two interstitial deletions on 1p36.3. Taken together, allelic loss of 1p was detected in 15 out of 22 (68%) oligodendrogliomas, 11 out of 14 (79%) anaplastic oligodendrogliomas, six out of 13 (46%) of oligoastrocytomas, and one out of three (33%) anaplastic oligoastrocytomas. Tumours with interstitial deletion on 1p36.3 constitute 12% (six out of 52) of all OTs examined.

### Determination of deletion breakpoints on 1p36.3

**Figure 3:**
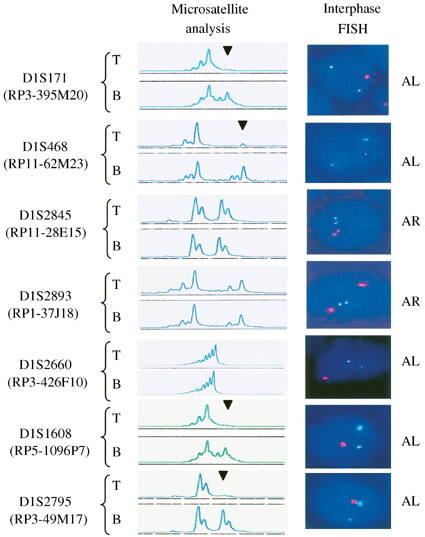
Representative results of microsatellite and interphase FISH analyses of case A18. Microsatellite markers are indicated on left with corresponding BAC or PAC clones containing such markers in parentheses. Concordant results are obtained for both techniques. Of note is that FISH reveals allelic loss at the noninformative locus D1S2660. Allelic loss (AL) in microsatellite analysis is indicated by arrowhead and is represented by one red (target) signal and two green (chromosome 1 centromere) signals in FISH. Allelic retention (AR) is indicated by the presence of both alleles in microsatellite analysis and is represented by two red and two green signals. T=tumour; B=tumour-matched blood.

We then employed an independent assay, interphase FISH, to verify the allelic status and to determine the deletion breakpoints in tumours that showed interstitial deletion. Four cases (A18, A19, B9 and B14), for which tissues were available, were subjected to FISH. Representative results of FISH are shown in Figure 3

**Figure 4:**
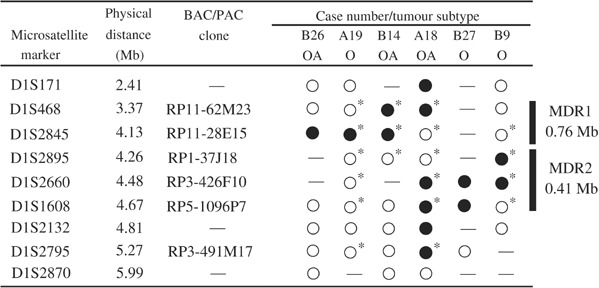
Delineation of MDRs on 1p36.3 in OTs by combined microsatellite and FISH analyses. FISH revealed the allelic status of six noninformative polymorphic markers (D1S2893, D1S2660 and D1S1608 in A19; D1S468 in B14; D1S2660 in A18; and D1S2893 in B9). The solid bars represent MDRs. O=oligodendroglioma WHO grade II; OA=oligoastrocytoma WHO grade II; filled circle=LOH; open circle=retention of heterozygosity; –=noninformative; ^*^ allelic status confirmed or revealed by FISH.

. Tumours were scored as having LOH when more than 70% of counted nuclei showed single target signal and two reference signals. Figure 4

summarises the allelic status, determined by both microsatellite and FISH analyses, of six OTs that showed interstitial deletion at 1p36.3. Concordant results were obtained for both techniques. Moreover, FISH revealed the allelic status of six more markers among the four tumours examined. Based on these data, two minimally deleted regions (MDRs) are delineated. MDR1 maps to a region of 0.76 Mb, defined by D1S468 and D1S2845, and MDR2 locates to an interval of 0.41 Mb, bound by D1S2893 and D1S1608.

### Genes annotated in MDRs

Based on the latest version of reference human genome sequence (Build 34, July 2003 freeze) the MDRs are covered by two sequence contigs (NT_004321 and NT_004547) with a gap of suggested size of 50 kb between these contigs. MDR1 starts from D1S468, which locates about 230 kb to the proximal end of NT_004321, spans over the sequence gap and ends at D1S2845, which is 480 kb from the distal end of NT_004547, whereas MDR2 lies in NT_004547. There are seven annotated genes clustered at the distal region of MDR1 on NT_004321 (Table 1

**Table 1:** A list of annotated genes mapped between D1S468 and D1S1608 on chromosome 1p36.3

**Locus**	**Gene^a^**	**Accession no.**	**Domain^b^**	**Biological function**
D1S468				
	TP73	NM_005427	p53	Transactivates p53 target genes, suppresses cell proliferation and induces apoptosis
			Sterile alpha motif	N-terminally truncated isoforms have anti-apoptotic functions
	KIAA0495	AB007964	Unknown	Hypothetical protein with unknown function
	FLJ32825	NM_152492	Chromosome segregation ATPase	Hypothetical protein with unknown function
	KIAA1185	NM_020710	Leucine-rich repeat	Hypothetical protein with unknown function
			Phenylalanyl-tRNA synthetase	
	KJAA0562	NM_014704	Unknown	Hypothetical protein with unknown function
	DFFB	NM_004402	CIDE-N domain	Involved in both DNA fragmentation and chromatin condensation during apoptosis
	LOC339448	XM_290902	Unknown	Hypothetical protein with unknown function
D1S2845				
D1S2893				
D1S2660				
	SHREW1	NM_018836	Transmembrane	Binds to E-cadherin and *β*-catenin in adherens junctions in polarised epithelial cells
D1S1608				

a Based on reference human genome sequence Build 34.

b Based on LocusLink (http://www.ncbi.nlm.nih.gov/LocusLink) and InterProScan (http://www.ebi.ac.uk/interpro).

). However, in a genomic interval of about 1 Mb covering the proximal region of MDR1 to end of MDR2, there is only one single gene identified, SHREW1.

### Allelic statuses of 1q and 19q

Two loci, D1S215 (1q25.2) and D1S2625 (1q31.2), were investigated for LOH on 1q arm. All tumours except case B24 were informative at either or both markers. Three tumours (A16, B1 and B7) had lost heterozygosity at both markers on 1q and also at all informative markers on 1p, suggesting that the entire chromosome was lost. Allelic loss of both 1q markers was also observed in tumour A19, which harboured interstitial deletion of 1p at D1S2845. B4 had lost heterozygosity at distal marker D1S2625 but maintained allelic balance at D1S215, whereas A25 showed LOH at D1S2625 and was noninformative at D1S215. Together, six out of 51 (12%) informative OTs exhibited LOH on 1q.

Two markers, D19S219 and D19S412, which are 1.02 Mb apart and locate in the common region of deletion on 19q13.3 were investigated for allelic loss. Out of 51, 33 (65%) informative tumours exhibited LOH in at least one of the 19q markers. In all, 10 tumours (A2, A5, A11, A18, A19, A22, A23, B13, B26 and B27) had lost heterozygosity at either marker but maintained allelic balance at the other marker on 19q.

Taken together, 29 out of 51 (57%) informative tumours exhibited LOH of at least one marker on both 1p and 19q. Three tumours (B6, B17 and B14) had 1p LOH in the absence of detectable 19q LOH, whereas four tumours (A9, A22, B10 and B20) exhibited 19q LOH but no 1p LOH.

### Statistical analysis

The 1p and 19q allelic status was correlated with clinicopathological parameters. 1p LOH was found to be closely associated with 19q LOH in OTs (*P*<0.0001). No statistical significance was observed between 1p LOH, 19q LOH or combined 1p/19q LOH and patient's gender, tumour type or grade. All six OTs with interstitial deletion on 1p36.3 were of WHO grade II, but only a trend was seen between interstitial deletion and tumour grade (*P*=0.065).

## DISCUSSION

Recurrent deletion of chromosome 1p in OTs strongly implies the presence of tumour suppressor gene(s) on this chromosome arm. With an aim to isolate the tumour suppressor gene by positional cloning approach, we set out to map the minimally deleted regions on the 1p arm. A series of 52 OTs were examined for allelic deletion by two rounds of microsatellite analysis followed by confirmation with FISH. Six tumours were found to harbour small interstitial deletion specifically on 1p36.3. Previous deletion mapping studies had revealed a few OTs with interstitial deletion on 1p, thus hindering a precise mapping of the critical deletion region (Husemann *et al*, 1999; Smith *et al*, 1999). This was likely due to the limited number of markers examined and to the wider genetic spacing of these markers. By examining more closely spaced markers on 1p36 in our microsatellite analysis, we were able to detect interstitial deletion in 12% of our tumour series. The proportion of tumours with interstitial deletion on 1p is likely to be an underestimation, as other previously defined deletion regions were not subject to high-resolution mapping as in this study. The small overlapping intervals on 1p36.3 have allowed us to delineate two contiguous MDRs, which fall in the overlapped region of deletion defined by Smith *et al* (1999) and Husemann *et al* (1999), providing further evidence that the 1p36.3 region is involved in tumorigenesis of OTs. Moreover, our deletion mapping study has narrowed down the critical deletion region on 1p36.3 to ∼1.2 Mb. This genomic region has been sequenced almost to entirety with a small sequence gap, for which no genomic clones are available. There are eight genes annotated in the MDRs (Table 1). Of these genes, only TP73, DFFB and SHREW1 have been studied previously.

The TP73 gene encodes product that shares significant structural and functional homology with the p53 tumour suppressor, raising the possibility that p73 might be a putative tumour suppressor (reviewed in Melino *et al*, 2002). Indeed, functional studies have revealed that p73 has antiproliferative and proapoptotic activity, independent of p53 status (Zhu *et al*, 1998a; Yuan *et al*, 1999). However, unlike TP53, somatic mutation of TP73 in human cancer is very rare (Mai *et al*, 1998; Alonso *et al*, 2001; Dong *et al*, 2002; Melino *et al*, 2002), suggesting that p73 does not act as a classical Knudson-type tumour suppressor. When transcript levels were examined, TP73 was found to be overexpressed in a variety of tumours (Tannapfel *et al*, 1999; Zaika *et al*, 1999), but its expression is reduced or absent in certain haematological malignancies (Corn *et al*, 1999; Kawano *et al*, 1999). Such downregulation of transcription is attributed to promoter hypermethylation (Corn *et al*, 1999; Kawano *et al*, 1999). In OTs, promoter hypermethylation of TP73 is detectable in 8–24% of tumours and anaplastic tumours are more common to harbour such aberration (Dong *et al*, 2001; Watanabe *et al*, 2002; Alonso *et al*, 2003). The TP73 methylation status has also been associated with decreased level of TP73 transcript (Dong *et al*, 2002). Taken together, these data suggest that p73 is involved in a subset of OTs. However, the recent discovery of N-terminal truncated isoforms of p73, namely ΔNp73, has provided new insights into the role of p73 plays in tumorigenesis. Functional studies showed that ΔNp73 is a transdominant inhibitor of wild-type p53 and p73 by counteracting their target gene transactivation, apoptosis and growth suppression and is able to immortalise primary mouse embryo fibroblasts *in vitro* (Grob *et al*, 2001; Nakagawa *et al*, 2002; Stiewe *et al*, 2002; Petrenko *et al*, 2003). These data demonstrate that ΔNp73 acts as an oncogene and has opposing activity of p73. Given the diverse function of p73 isoforms, it is thus of paramount importance to resolve the functional contribution of each isoform toward cancer development.

DFFB is the other known gene identified in MDR1. It encodes the caspase-activated DNase and is responsible for degradation of chromosomal DNA during apoptosis. Its gene product is complexed with an inhibitor encoded by DFFA *in vivo*. Upon apoptosis induction, caspase cleaves the inhibitor and releases DFFB from the complex to enter into nuclei to degrade chromosomal DNA (Nagase *et al*, 2003). No somatic mutations of DFFB were detected in 41 neuroblastomas examined (Judson *et al*, 2000). However, aberrant DFFB transcripts resulting from splicing have been reported in hepatocellular carcinoma (Hsieh *et al*, 2003).

SHREW1 appears to be the single gene located in MDR2. Recently, the cDNA of SHREW1 was isolated from invasive endometriotic cells (Bharti *et al*, 2004). This gene encodes a ∼48kDa transmembrane protein, with its carboxy terminus being cytoplasmic, and does not belong to any protein family. Ectopic expression of SHREW1 in polarised epithelial cells showed that shrew1 protein colocalised with E-cadherin in adherens junctions, probably via binding of *β*-catenin. However, in nonpolarised invasive epithelial cells, shrew1 bound neither to N-cadherin and *β*-catenin (Bharti *et al*, 2004). E-cadherin is generally not expressed in brain tumours, except in benign meningiomas (Schwechheimer *et al*, 1998), whereas N-cadherin and *β*-catenin are detectable at cell–cell junctions in malignant astrocytic tumours (Utsuki *et al*, 2002). The N-cadherin and *β*-catenin status in OT is not known. Further investigation of SHREW1 function may shed light on the role of this gene plays in cancer development and its involvement in invasion.

In conclusion, our deletion mapping study has refined one of the candidate tumour suppressor loci on chromosome arms 1p in OTs. Two contiguous MDRs are delineated from a small interval on 1p36.3, between D1S468 and D1S1608, which contain only eight known and novel genes. These candidate genes are currently under investigation for their involvement in OTs.

## Note added in proof

A recent report by Felsberg *et al* (*Brain Pathology* 2004; **14**: 121–130) marked one of the commonly deleted regions to 1p36.31-pter in oligodendroglial tumours. Their results are in line with our findings.
